# Clinical application prospect of umbilical cord-derived mesenchymal stem cells on clearance of advanced glycation end products through autophagy on diabetic wound

**DOI:** 10.1186/s40001-017-0253-1

**Published:** 2017-03-24

**Authors:** Yanfu Han, Tianjun Sun, Ran Tao, Yanqing Han, Jing Liu

**Affiliations:** 1 Department of Plastic Surgery, Beijing Shijitan Hospital, Capital Medical University, 10 Tieyilu, Yangfangdian, Haidian District, Beijing, 100038 People’s Republic of China; 2 Department of Burn and Plastic Surgery, Hainan Branch of People’s Liberation Army General Hospital, Haitangwan, Sanya, People’s Republic of China; 3 Department of Plastic Surgery, PLA General Hospital, 28 Fuxing Road, Beijing, People’s Republic of China; 4 School of Electrical and Information Engineering, Wuhan Institute of Technology, 366 Huquan, Wuhan, People’s Republic of China

**Keywords:** Mesenchymal stem cells, Diabetic wound, Advanced glycosylation end products, Wound healing, Application

## Abstract

Nowadays, wound healing delay due to diabetes is considered to be closely related to the accumulation of advanced glycation end products (AGEs). Although mesenchymal stem cells (MSCs) exhibit positive effects on diabetic wound healing, related mechanisms are still not fully elucidated. It has been reported that MSCs can improve the activity of autophagy in injured tissues, thereby playing an important role in wound healing. The autophagy induced by MSCs may be beneficial to diabetic wound healing via removing AGEs, which provide new ideas for clinical treatment of diabetic wounds with the potential of broad application prospects. In this study, the current research situation and application prospect of umbilical cord-derived MSCs on the clearance of AGEs in diabetic wound were reviewed.

## Background

Currently, the incidence of diabetes is increasing year by year. A high morbidity of diabetic foot ulcers (more than 25%) is always found in diabetic patients [1]. As a common complication of diabetes, chronic diabetic wounds with the characteristics of low cure rate and high amputation rate not only causes great pain to patients, but also increases the burden of the family and society. Despite the existing therapy methods, there are still no effective therapy measures available for the treatment of diabetic wounds owing to its complex mechanisms and characteristics. Therefore, it is urgent to search for new approaches to promote wound healing in diabetic patients.

The mechanisms of diabetic wound healing are complicated. It has been shown that the mechanisms of diabetic wound healing are associated with various factors, including ischemia and hypoxia, abnormal inflammatory response, excessive microbial load, peripheral neuropathy, and abnormal expressions of matrix metalloproteinases and vascular endothelial growth factors. However, clear mechanisms of diabetic wound healing have not been fully clarified. With the deepening study on pathogenesis of diabetic skin lesions, advanced glycation end products (AGEs) have been revealed to be closely related to the delayed healing of diabetic skin wounds [2]. AGEs are the final products of non-enzymatic catalytic reaction of amino (proteins, lipids, and nucleic acids) and aldehyde groups of carbohydrate under continuous high level of glucose conditions. In patients with diabetes, pathologic high blood glucose could always accelerate the glycosylation reaction, thereby producing a large amount of AGEs. However, the accumulation of AGEs cannot be recovered to normal level by reducing blood glucose. AGEs, as the direct products of diabetic metabolic remodeling, are an important environmental medium for changes of skin tissue cells, extracellular matrix, and cytokines. Therefore, accumulation of AGEs could induce the occurrence of damaged skin in diabetic patients. Besides, AGEs also exhibit the ability to inhibit fibroblast differentiation, proliferation, and migration, which could delay the process of wound healing through decreasing the expression of collagen [3]. Some animal experiments have suggested that the application of anti-AGE agents could promote the healing of soft tissue wounds [4]. Although the accumulation of dermal AGEs has been considered as a fundamental cause of diabetic wound formation or refractory healing, only few effective methods have been revealed to be able to inhibit and remove AGEs in the wound so far [5, 6].

## Research status

Recently, some positive effects of cathepsins on clearance of AGEs were revealed in vivo. Reported as follows, Grimm et al. [7] have demonstrated that cathepsin D plays an important role in the degradation of intracellular AGEs. Meanwhile, a further investigation of them has also shown that both cathepsin D and L have significant positive roles in the reducing of toxicity caused by AGEs [8]. Moreover, phytopharmaceutical Dispo85E is also reported having the ability to remove AGEs through the autophagy-lysosomal pathway induced by hepatocyte growth factor both in vivo and in vitro [9]. As an important protective mechanism in eukaryotic cells, autophagy plays a crucial role in the maintaining of homeostasis in the intracellular environment. During this process, autophagosome is firstly formed by packaging the cytoplasmic proteins and organelles into a double membrane, and then combined to the lysosome to form an autolysosome. Based on this autolysosome, the expressions of cathepsins will be obviously induced in lysosome, while the activity of lysosome is activated. Since autophagy is able to degrade toxic protein aggregation, damaged mitochondria, and other cellular organelles in cells [10], the accumulation of AGEs, a production of glucose metabolism, may also be removed by autophagy/cathepsin pathway. Nowadays, several small-molecule activators involved in autophagy pathways such as rapamycin, trehalose, lithium, and rilmenidine have been identified to be able to up-regulate autophagy activity and promote the degradation of the polyproteins. However, the application of these factors is still limited in the clinical setting. For example, the common autophagy inducer rapamycin is a major regulatory protein in insulin signaling pathway, which could also influence many other metabolic pathways. Meanwhile, the autophagy induction process of rapamycin is relatively slow with a long indirect acting time, and the effects of rapamycin were always temporary [11]. Therefore, searching for new ideal autophagy inducer exhibited important application values in the clinical setting.

Until now, there are still some patients with diabetic wound failed to cure in spite of continuous development on the treatment technology of diabetic wounds [12]. Recently, the advent of stem cell therapy, which is considered to be the basis of tissue regeneration, has brought great hope to these diabetic patients [13]. Mesenchymal stem cells (MSCs) are a kind of tissue stem cells with multiple differentiation potential, which are initially found in bone marrow. The promotion effect of different sources of MSCs on diabetic wound healing has been investigated by several researchers. Local application of allogeneic bone marrow-derived MSCs could promote wound healing in diabetic rats [14]. Meanwhile, adipose tissue and hair follicle dermal sheath-derived MSCs are also beneficial to chronic wound healing in patients with diabetes [15, 16]. Moreover, compared with bone marrow-derived MSCs, umbilical cord-derived MSCs (UCMSCs) possess better biological characteristics such as easy obtainability, rich source, no ethical problems, and strong proliferation potential and show effective promotion effect on wound healing of skin ulcer in patients suffered with type 2 diabetes [17]. Further research has indicated that positive effects of UCMSCs on wound healing were associated with differentiation into keratinocyte and release of ICAM-1, TIMP-1, and VEGF-A [18]. Although the accumulation of AGEs has been identified to be one of the fundamental causes of impaired wound healing in diabetes [5], the scavenging effects of UCMSCs on AGEs and its related molecular mechanism are still elucidated.

Preliminary results on the relations between autophagy and wound healing have been shown by various studies. As reported, the expression of autophagy-related gene Beclin-l in burn wound was significantly increased, and increased autophagy was found to be helpful to wound healing [19]. However, excessive autophagy could induce apoptosis and result in delay in wound healing [20]. In addition, similar to apoptosis and necrosis, autophagy also serves a vital role in the process of deepening wound [21, 22]. It has been reported that adenosine could promote wound healing of diabetic ischemic ulcers through improving autophagy of endothelial progenitor cells [23]. MSC transplantation was beneficial to carbon tetrachloride-induced liver regeneration and survival of pulmonary epithelial cells in acute lung injury through autophagy upregulation [24, 25]. MSCs are also considered to be able to alleviate beta cell damage induced by chronic high glucose via regulation of autophagy both in vivo and vitro [26]. All the above researches have illustrated that enhanced autophagy activity is favorable in the regeneration and reparation of injured tissue cells. On the other hand, MSCs are also reported being able to activate autophagy, thereby removing intracellular accumulation of toxic proteins and increasing the viability and survival of neurons [27]. Meanwhile, MSC-induced autophagy could also remove abnormal proteins or amino acids in vivo and promote cell repair. However, the mechanisms and promotion effects of MSCs on autophagy activity and scavenging capacity in diabetic wounds are still unclear. Hence, research on the clearance effects of autophagy induced by MSC transplantation in wounds and its related molecular mechanism will become a hot topic and urgently need to be concerned.

Diabetic wounds are not only accompanied by the accumulation of high glucose and AGEs, but also associated with ischemia and hypoxia. Under anoxic condition, the dissociation of Beclin-1 from Bcl-XL and Bcl-2 could be induced by activated hypoxia-inducing factor 1α (HIF-1α) through upregulated BNIP3, and then the released Beclin-l could promote autophagy via serving as a positive regulator in this procedure. HIF-1α/BNIP3/Beclin-1 has been considered to be an important signaling pathway in the upregulation of autophagy under hypoxia [28]. The expression of HIF-1α is significantly reduced in diabetic skin wounds [29], while MSCs have been found to promote wound healing through upregulating HIF-1α in fibroblast cells [30]. Therefore, we suspected that the wound healing might be improved by MSC transplantation-induced autophagy via the HIF-1α/BNIP3/Beclin-1 pathway and the clearance of AGEs through autophagy/cathepsin.

## Application prospect

To sum up, autophagy/cathepsin is able to enhance cell viability and proliferation ability through clearance of intracellular AGEs. As a new approach to promote wound healing, UCMSC application has certain safety and no side effects, as well as a good ability in clearing AGEs via enhancing autophagy; therefore, UCMSCs may become an ideal autophagy inducer in the promotion of wound healing and provide new ideas and strategies for the treatment of diabetic wound healing in the clinical setting. However, the popularization and application of further clinical research findings are still required to further confirm the effects of this method in increasing wound healing rate, reducing amputation rate, hospital stay and costs, relieving the pain and improving the life quality of diabetic patients with chronic wounds, as well as reducing the burden of family and society. At present, with a high prevalence of diabetes, the rate of diabetic patients complicated with chronic ulcer wound is grown to 25%. The clinical application of UCMSCs in the treatment of diabetic wounds will bring out the significant economic and social benefits with a broad prospective.
